# Presence of Anti-Glomerular Basement Membrane Antibodies and Myeloperoxidase Anti-Neutrophilic Cytoplasmic Antibodies in a Case of Rapidly Progressive Glomerulonephritis

**DOI:** 10.3389/fmed.2015.00053

**Published:** 2015-08-07

**Authors:** Gaurang P. Mavani, Max Pommier, Sandar Win, Michael F. Michelis, Jordan Rosenstock

**Affiliations:** ^1^Division of Nephrology, Department of Medicine, Lenox Hill Hospital, New York, NY, USA

**Keywords:** RPGN, anti-MPO antibody, anti-GBM antibody, plasmapheresis, cyclophosphamide

## Abstract

A 69-year-old male had initially presented with low-grade proteinuria, microhematuria, and a positive myeloperoxidase anti-neutrophilic antibody (ANCA). He subsequently developed deterioration of kidney function and developed uremic symptoms. Creatinine was 486.2 μmol/L (5.5 mg/dL). Anti-MPO was positive (titer >8 U, normal <0.4). He was clinically diagnosed with rapidly proliferative glomerulonephritis most likely due to ANCA vasculitis. He received three doses of pulse methylprednisolone therapy. Kidney biopsy showed pauci-immune glomerulonephritis. Immunofluorescence was positive for faint linear IgG staining of glomerular basement membrane (GBM). Anti-GBM antibody was positive 2.1 U (normal <1). He was started on high-dose oral steroids; monthly intravenous cyclophosphamide and plasmapheresis were also initiated. His symptoms improved and creatinine is 247.5 μmol/L (2.8 mg/dL). His repeat anti-GBM antibody was negative. This is a rare case of rapidly progressive glomerulonephritis due to dual MPO-ANCA antibodies and anti-GBM antibodies (DAV).

## Introduction

Vasculitis secondary to the combination of anti-glomerular basement membrane (GBM) antibody and ANCA is rare. Depending upon the series studied, the incidence is 0.47–0.55 per million people per year (1). Several other studies have also studied the association between ANCA and anti-GBM antibodies. Depending upon the series between 5 and 14% of patients with primarily ANCA vasculitis also were found to have anti-GBM antibodies and between 30 and 38% of patients with anti-GBM disease also had ANCA antibodies (2, 3). Light microscopy in patients with DAV cannot be differentiated from patients who had ANCA or anti-GBM vasculitis alone (4).

In this case report, we present a case of rapidly proliferative glomerulonephritis (RPGN) who had DAV who responded to treatment.

## Case Summary

**Figure 1:**
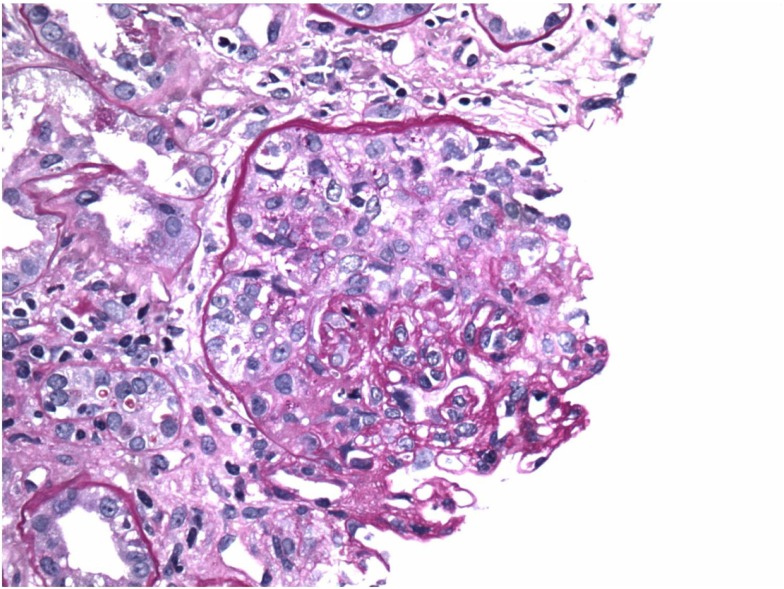
**Crescents on light microscopy**.

**Figure 2:**
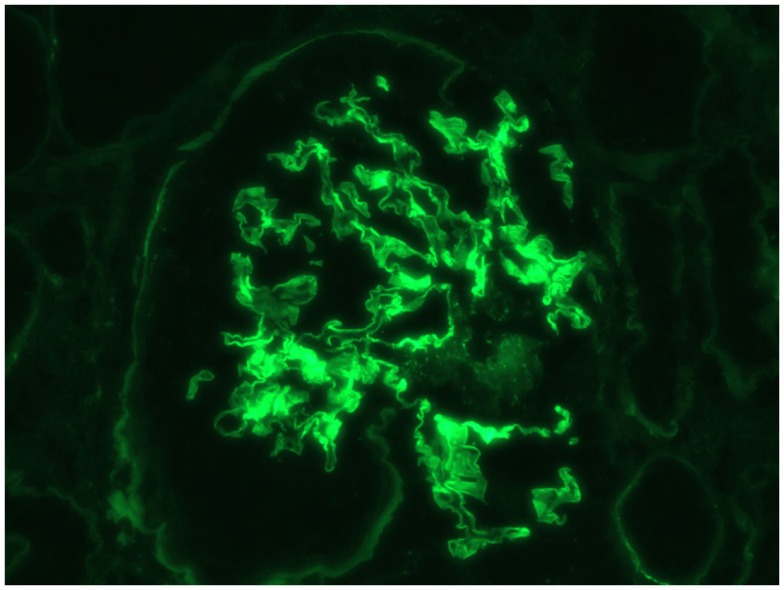
**Immunofluorescence linear staining of IgG**.

This is a 69-year-old male with a history of diabetes mellitus, hypertension, and no history of kidney disease was referred to the nephrologist for evaluation of microscopic hematuria, proteinuria, and borderline elevated creatinine. He previously had a negative cystoscopy. He was asymptomatic on presentation. His home medicines included metoprolol, enalapril, simvastatin, metformin, and aspirin. His initial work-up revealed creatinine of 117.57 μmol/L (1.33 mg/dL). UA was positive for RBC and protein. Urine protein/creatinine ratio was 728 mg/g. MPO-ANCA was positive >8 U (Normal <0.4). Proteinase 3b and ANA were negative. Serum complements were normal; hepatitis panel was negative. Kidney biopsy was advised but he refused. He agreed to monthly follow-up, but was non-compliant with his follow-up. He presented to ER after 3 months with vomiting, weakness, and decreased urine output. Creatinine was 461.44 μmol/L (5.22 mg/dL) and his urine showed RBC, RBC cast, and was positive for protein. It was felt that he likely had RPGN secondary to ANCA vasculitis. He was hospitalized and intravenous fluid was initiated. Aspirin and metformin were held. He was given kayexalate and was given 1 g of pulse methypredinisolone therapy daily for 3 days. He had a kidney biopsy, which showed diffuse necrotizing, sclerosing, and primarily pauci-immune crescentic glomerulonephritis (Figure 1). There were no changes of diabetic nephropathy. Immunofluorescence also revealed faint linear staining of GBM for IgG (Figure 2). Due to linear GBM staining anti-GBM antibody was sent, which was positive (2.1). Three doses of pulse methylprednisolone therapy were followed by oral prednisone 100 mg po daily and intravenous cyclophosphamide was initiated. A tunneled catheter was placed and he had five plasma exchanges over 2 weeks. The most recent serum creatinine was 2.0 mg/dL. Repeat anti-GBM antibody was negative.

## Discussion

Circulating ANCA and anti-GBM are both associated with rapidly proliferative glomerulonephritis. Both are rare diseases. ANCA is usually present in older patients with prevalence of 2.5/100,000. Anti-GBM disease is still rarer with prevalence of 0.5–0.9/1,000,000 (5). As mentioned before, the incidence of DAV is rarer but still can occur up to 14% in patients with primarily ANCA and in up to 38% of patients with primarily anti-GBM disease (3). ANCA vasculitis is a very complex disorder, which has been linked to infections, silica, mercury, lead, and drugs like propylthiouracil, allopurinol, and hydralazine. Most cases, however, are idiopathic (6). ANCA antibodies can attach to antigens present with in neutrophil granules, causing degranulation of the neutrophils thus causing damage due cytotoxic oxygen free radical species, so ANCA antibodies are thus not only a marker of the disease but may also be directly pathogenic (7). The target for anti-GBM antibody is alpha 3 chain of type 4 collagen on GBM and alveoli (5). Renal involvement in both type of vasculitis is usually crescentic necrotizing glomerulonephritis. Immunofluorescence in anti-GBM disease reveals very distinct strong linear basement IgG staining of GBM, while in ANCA vasculitis immunofluorescence is negative (2, 5). In this case, immunofluorescence of kidney biopsy specimen revealed only faint linear deposit of IgG on the GBM.

There are important clinical implications of having dual antibody positive as opposed to only one in terms of clinical presentation, prognosis, relapse, and treatment.

Systemic complaints are common in ANCA vasculitis (8). In anti-GBM antibody disease, systemic symptoms are very rare and usually present with end organ involvement like pulmonary hemorrhage and RPGN. Thus, when anti-GBM disease patients have systemic symptoms earlier in the course, it may indicate that patient may have concurrent DAV (9).

Relapse is not uncommon in ANCA vasculitis and can occur in 17% of cases. Relapse mostly occurs in 12–18 months after the initial remission. Anti-GBM antibody rarely recurs since antibody production is short lived. Relapse with DAV is higher than in anti-GBM disease (9).

Prognosis in DAV may be intermediate between isolated ANCA diseases and isolated anti-GBM disease. Prognosis in pure ANCA vasculitis has better prognosis compared to the other two. In one study, 75% of patients on dialysis due to pure ANCA-related disease recovered function with appropriate treatment (10). Bosch et al. showed that 45% of patients on dialysis due to DAV recovered significant renal function with treatment. None of the anti-GBM disease patients on dialysis was able to come off dialysis (11). The best predictor of outcome in anti-GBM disease is the severity of glomerular injury, creatinine at presentation, and titer of anti-GBM antibody (9). Rutgers et al., however, found DAV prognosis more similar to anti-GBM disease alone. He reported that dialysis dependency at admission was 28% in MPO–ANCA positive patients, 60% in DAV, and 69% with anti-GBM disease patients. Cumulative renal survival at 1 year was 64% for MPO–ANCA vasculitis, 10% for DAV, and 15% for anti- GBM disease. None of their patients on dialysis recovered kidney function (4). Levy et al. noted that patients with DAV had poor response to therapy and their prognosis was similar to patients with pure anti-GBM disease inspite of receiving plasmapheresis in addition to immunosuppression (12).

Treatment of vasculitis due to ANCA antibody is cyclophosphamide or rituximab and steroids (13). Plasmapheresis is sometimes added when renal failure is severe or rapidly progressing (14). Treatment of anti-GBM disease is plasmapheresis, cyclophosphamide, and steroids. Treatment of DAV is the same as in anti-GBM disease (12).

There were some atypical features of this case that are worth emphasizing. Systemic ANCA vasculitis typically presents with constitutional symptoms, such as fever, malaise, arthralgia, and weight loss. Prodromal symptoms can last for a few months before the involvement of a specific organ (15). In a study of 70 patients with ANCA vasculitis, isolated renal vasculitis was noted in 25% of patients. Patients with isolated renal vasculitis had advanced glomerulosclerosis as they did not have any constitutional symptoms and presented late in the course of the disease (8). Our patient presented to us early in the course of his disease, with minimally elevated creatinine, microhematuria, and proteinuria without any constitutional symptoms and only later developed uremic symptoms when kidney function deteriorated. As this patient initially presented with minimal disease, one could have attributed this to diabetic kidney disease, and argued that checking ANCA initially in a patient without RPGN and no symptoms was unnecessary. Studies have shown that microhematuria can be seen in up to 30% of patients with diabetic nephropathy (16). However, a Japanese study in 154 patients with diabetes mellitus found that 7% of patients with microhematuria had glomerular diseases other than diabetic nephropathy. Also, patients who had diabetic nephropathy and microhematuria were more likely to have advanced diffuse, nodular, exudative, and interstitial lesions than those in the non-hematuria group (17). So the presence of microhematuria in a diabetic patient even without a typical presentation of vasculitis-like RPGN should trigger workup for non-diabetic glomerulopathy, such as vasculitis, especially if the patient is not known to have established diabetic nephropathy.

Furthermore, immunofluorescence microscopy in this patient showed very faint linear IgG staining of the GBM, which is not typical of anti-GBM disease. Immunofluorescence microscopy in anti-GBM disease typically shows very dense, strong staining of GBM (2). Faint linear staining of GBM can be a feature of diabetic nephropathy. Our patient was diabetic, but there were no other features of diabetic nephropathy on the biopsy. It is rare to have linear staining due to diabetes in the absence of light microscopy evidence of diabetic nephropathy (18). Thus, the presence of any linear staining of GBM in the setting of glomerulonephritis, in the absence of light microscopy evidence of diabetic nephropathy, should suggest the presence of anti-GBM disease.

## Conclusion

Patients who have either ANCA related disease or anti-GBM-related disease should be tested for other antibodies as both these antibodies are sometimes present together. Presentation, treatment, and prognosis of dual antibody vasculitis may be different from isolated ANCA related vasculitis. Isolated renal vasculitis should be considered in an asymptomatic patient who presents with proteinuria and microhematuria. Also, even faint linear staining without the presence of diabetic nephropathy can suggest anti-GBM disease.

## Conflict of Interest Statement

The authors declare that the research was conducted in the absence of any commercial or financial relationships that could be construed as a potential conflict of interest.

## Author’s Note

Consent has been obtained from the patient for publication of this case report.
